# Comprehensive review of myocardial injury after noncardiac surgery: prevention, intervention, and long-term management strategies

**DOI:** 10.1186/s13019-025-03358-1

**Published:** 2025-01-30

**Authors:** Moiud Mohyeldin, Sarah J. Norman, Ayzia Carney, Courtney Odza

**Affiliations:** 1 Faculty of Medicine, UMST University, Khartoum, Sudan; 2 School of Medicine, American University of the Caribbean, Cupecoy, Sint Maarten; 3 School of Medicine, American University of the Caribbean, Cupecoy, Sint Maarten; 4 School of Medicine, American University of the Caribbean, Cupecoy, Sint Maarten

**Keywords:** Myocardial Injury, Noncardiac surgery, Prevention, Management, Cardiovascular Risk

## Abstract

Myocardial Injury after Noncardiac Surgery (MINS) is an increasingly recognized complication that significantly impacts postoperative morbidity and mortality. Characterized by elevated cardiac troponin levels without overt ischemic symptoms, MINS presents a challenge in perioperative care. This review article explores the epidemiology, etiology, and management of MINS, with a particular focus on prevention and the latest management strategies. We discuss the role of aspirin, statins, anticoagulation, and Dual Antiplatelet Therapy (DAPT) within the context of MINS, drawing on evidence from notable clinical trials as well as observational studies. Despite advancements in understanding and managing MINS, the condition continues to be associated with high mortality and major adverse cardiovascular events (MACE), underscoring the need for ongoing research and development of more effective management protocols.

## Introduction

Myocardial Injury after Noncardiac Surgery (MINS) is a clinical condition marked by elevated cardiac troponin levels following noncardiac surgery, which may occur with or without ischemic symptoms. Since its first detailed description in 2014, MINS has been associated with increased short- and long-term mortality occurring in roughly 8% of cases, making it a significant concern for clinicians [1]. The number of patients undergoing surgery has significantly increased from twenty years ago, and with commonly asymptomatic presentations of MINS, many cases can go undetected each year. Therefore, primary intervention and management will be the determining factors for increased survival rates. Furthermore, intraoperative and post-operative costs of hospitalized patients with complications continue to climb annually [2]. Preemptively identifying and managing at-risk patients can have a significant impact on hospital expenditures [2]. In this literature review, the sequence of events leading up to the diagnosis is discussed along with the diagnostic features to evaluate in patients. While there is no direct cause of this condition, MINS continues to gain traction in the medical community.

### Etiologies of myocardial injury after noncardiac surgery

Understanding the various etiologies of MINS is critical for prevention and perioperative interventions. The disease etiology of MINS has been theorized to be related to myocardial infarction caused by plaque rupture or oxygen-supply demand mismatch, and extracardiac causes [3]. For example, a study by Puelacher and associates assessed 848 out of 5,602 patients undergoing noncardiac surgeries that developed MINS to determine potential causalities [4]. These patients underwent testing to conclude the cause of MINS, including coronary angiography, electrocardiogram (ECG) testing, and cardiac biomarker evaluation. In the context of this study, type 1 myocardial infarctions (caused by thrombosis of ruptured arterial plaque) are further subcategorized into tachyarrhythmias or acute heart failure (AHF), type 2 myocardial infarctions (resulting from an imbalance between oxygen supply and demand) with documented anemia or hypotension, and extracardiac causes such as pulmonary embolism, cardiac trauma, or sepsis [4]. Causes of MINS for this study were attributed to all three etiological categories, 71.7% from type 2 myocardial infarction, 15.1% from type 1 myocardial infarction, and 13.1% due to extracardiac causes [4]. Overall, these disease etiologies are associated with MACE and increased mortality in the setting of MINS.

Literature suggests that MINS can occur from any combination of pre-existing medical conditions, perioperative stress, inflammation, hemodynamic changes, and ischemia-reperfusion injury during surgery. Patients with underlying cardiovascular conditions, such as coronary artery disease, hypertension, or heart failure are at an increased risk for developing MINS due to compromised myocardial reserve and increased susceptibility to intraoperative ischemic events [5]. Physiological stress responses, including increased sympathetic activity, catecholamine release, and hemodynamic changes can be induced by surgical procedures. This can lead to increased myocardial oxygen demand and precipitate myocardial injury in vulnerable patients [3]. Furthermore, surgical trauma can induce systemic inflammatory responses causing the release of pro-inflammatory cytokines and immune cell activation. Excessive inflammation can then disrupt endothelial function, promote plaque instability, and contribute to microvascular dysfunction [4]. Additionally, perioperative hemodynamic fluctuations, caused by hypotension or arrhythmias, can compromise myocardial perfusion and oxygen delivery further increasing the risk of myocardial ischemia and subsequent injury. Lastly, during surgery, reperfusion of ischemic myocardial tissue can induce oxidative stress, calcium overload, and inflammatory responses [5]. Understanding these underlying mechanisms is critical for implementing preventative strategies and optimizing preoperative and postoperative care to mitigate the risk of myocardial injury in surgical patients.

**Fig. 1:**
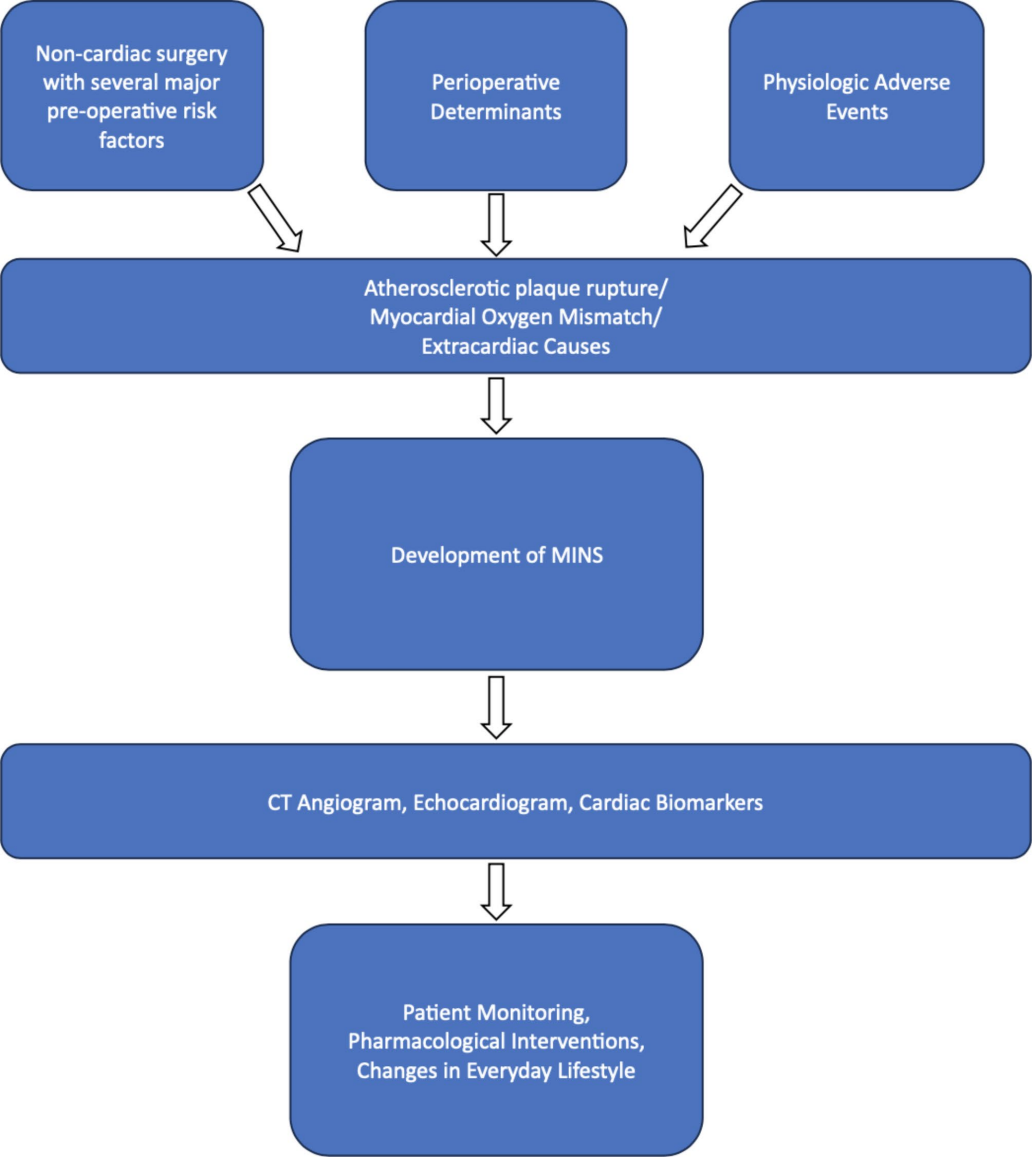
The development of MINS

Other risk factors for developing MINS include individual patient characteristics such as elderly patients, those with a prior medical history of cardiovascular adverse events, and the presence of other comorbidities [6]. Furthermore, surgical factors such as type of surgery and duration, and intraoperative events such as blood loss, anesthetic agents, SIRS, and perioperative infections can all contribute to the development of MINS. Per the various disease etiologies, preexisting medical conditions, and surgical factors, postoperative patients with MINS have a twofold risk of mortality compared to postoperative patients without MINS even years after follow-up [6]. Figure 1 illustrates the etiology and pathophysiology of MINS as well as workup and management.

### Diagnosis of myocardial injury after noncardiac surgery

MINS remains a diagnosis of exclusion, but the criteria has been re-evaluated to include a broader definition in the hopes of avoiding otherwise undetected cases. Botto et al. conducted a large, international prospective cohort study proposing a diagnosis of MINS made without the requirement of a presenting ischemic symptom but as a peak in Troponin T levels of 0.03ng/ml or greater secondary to myocardial ischemia within 30 days following surgery [7]. This criterion is a major distinguishing factor as the Perioperative Ischemic Evaluation (POISE) trial found as many as 65% of 415 patients with MINS did not experience any ischemic symptoms [7]. Of note, it is also important to consider that many patients do not fall into the classic, Type I myocardial infarction category defined by atherosclerotic plaque rupture and subsequent thrombosis; however, some patients may experience myocardial infarct secondary to oxygen demand-supply mismatch. This mismatch can be induced by various causes and possibly augmented by underlying coronary artery disease though that is not always the case [8].

Invasive and non-invasive imaging modalities have not been more successful in terms of predicting MINS but are the least cost-effective approach. There are markers of early detection that include preoperative cardiac troponin, N-terminal pro-B-type natriuretic peptide (Nt-ProBNP), and growth determination factor- 15 (GDF-15) which are a better cost alternative to diagnose MINS [9]. Nt-ProBNP was measured in blind Vascular Events in Noncardiac Surgery Cohort Evaluation (VISION) study that showed promising results when it came to determining perioperative risk in patients. Interestingly, a study performed by Meister found pre-operative cardiac troponin as the gold standard biomarker because it is cost-effective and can immediately deliver results [10]. VISION also dedicated a separate study identifying GDF-15 as a sensitive biomarker in patients around the time of surgery to help reduce the risk of mortality; however, NtPro-BNP is used more widely than GDF-15 as it is currently more accessible [9].

## Prevention

The prevention of MINS is critical for healthcare providers to decrease the occurrence of serious complications, reduce mortality rates, and improve patient outcomes while enhancing healthcare quality. To adequately impart preventative measures, medical institutions will need to implement the use of preoperative risk assessment tools, optimize cardiovascular risk factors, and utilize pharmacological and non-pharmacological interventions.

### Preoperative risk assessment tools

Preoperative risk assessment tools play a crucial role in identifying high-risk patients, personalizing care plans based on patient histories and risk factors, and assisting with providing comprehensive, standardized evaluations. The most useful preoperative risk assessment tools for MINS include the Revised Cardiac Risk Index (RCRI), American College of Surgeons National Surgical Quality Improvement Program (ACS NSQIP), Gupta Perioperative Risk for Myocardial Infarction or Cardiac Arrest (MICA), and VISION risk calculators.

The RCRI calculator is used to assess the risk of patients over forty-five years of age undergoing cardiac complications during noncardiac surgery. This tool evaluates six main parameters which include high-risk noncardiac surgery (intraperitoneal, intrathoracic, suprainguinal vascular surgeries), history of ischemic heart disease, congestive heart failure, prior transient ischemic attack or stroke, pre-operative treatment with insulin, and a preoperative creatinine over 2 mg/dL. The RCRI calculator further stratifies patients into four risk classes and estimates the risk of mortality within 30 days of noncardiac surgery. For patients with a score of one or greater for the RCRI calculator, providers can determine the appropriate postoperative cardiac monitoring (e.g., ECG, troponins). While this tool can be useful for determining the risk of MINS, there is limited predictive accuracy due to the assessment developing from retrospective data [11]. Additionally, RCRI does not adequately address all risk factors that could impact patient outcomes. According to a large systematic study conducted by Ford and associates, it was determined that the RCRI assessment was useful for stratifying patients of low risk versus high risk, but it was not useful in predicting cardiac events or death during vascular noncardiac surgeries [11].

The ACS-NSQIP risk assessment calculator was derived from extensive data collected from more than one million surgical cases and is considered an evidence-based risk prediction. The calculator assesses over 135 variables for preoperative patients undergoing surgical procedures and supplies the medical provider with a customized risk assessment. Healthcare facilities use the data acquired by the ACS-NSQIP tool to benchmark performance against national standards providing the framework to initiate improved initiatives and preoperative risk assessment optimization. It has been indicated that participating institutions can reduce postoperative complications by 45% and postoperative mortality by 27%^12^. While this assessment tool has specificity to the surgical population and extensive validation, the ACS-NSQIP risk calculator may have surgical population bias, generalizability, and limited predictive power due to the complexity of real-life clinical scenarios [12].

The Gupta Perioperative Risk for Myocardial Infarction or Cardiac Arrest (MICA) has proven valuable in clinical practice for accurately predicting MINS risk and providing a comprehensive assessment of preoperative variables, comorbidities, and other surgical factors. MICA formulates a risk factor score by incorporating age, functional status, ASA class, renal function, and type of surgical procedure. The MICA score has higher discriminatory power and predictive accuracy compared to the RCRI score, therefore MICA may be more effective at identifying high-risk patient populations [13]. Furthermore, the MICA assessment tool is beneficial for identifying patients at risk for developing MINS by taking into account risk factors and markers related to cardiac injury. Furthermore, Fronczek and associates claimed that the RCRI tends to overestimate risk in lower-risk patients, while MICA may be more accurate in predicting MINS for lower-risk patients [13].

The VISION risk calculator was derived from the VISION cohort study led by Duceppe and associates. This study sought to determine the relevancy of the predictive value of preoperative NT-proBNP in surgical patients who develop cardiac injury after undergoing noncardiac surgery [14]. The VISION study assessed data from over ten thousand patients from nine different countries by collecting preoperative NT-proBNP levels and troponin T levels daily for up to three days after surgery [14]. The purpose of this study was to address the lack of data on perioperative and postoperative cardiovascular events, including MINS, and to create another risk assessment tool to further guide clinicians in assessing cardiovascular risks in noncardiac surgical patients. Therefore, the VISION risk assessment takes into account NT-proBNP and the risk factors assessed by the RCRI tool to provide providers with advanced accuracy of predicting cardiovascular injury within thirty days after noncardiac surgery [14].

Each tool has its advantages and disadvantages when identifying cardiac complications postoperatively. When utilizing a risk assessment tool, there is variability in patient risk due to various populations and various study parameters [15]. For risk assessment tools to be useful, these calculations must identify higher-risk patients who will benefit from pre-operative cardiac optimization which may call for the use of numerous risk assessment types. However, it is imperative to understand the limitations of each risk assessment calculator used in MINS to address accuracy concerns and the potential overestimation or underestimation of risk in certain populations [15]. Overall, each preoperative patient must have a personalized care plan that takes into account medical history, lifestyle choices, comorbidities, and risk factors when utilizing risk assessments to provide more accurate predictions and recommendations for patient care.

### Optimization of cardiovascular risk factors

Healthcare providers can optimize cardiovascular risk factors for MINS through a preoperative assessment, in which clinicians will review the patient’s medical history, physical examination findings, and diagnostic tests to identify cardiovascular risks. The treatment of other comorbidities that contribute to cardiovascular risk such as obesity, sleep apnea, and chronic kidney disease are all imperative to reducing the risk of patients developing MINS [16]. For cardiovascular concerns specifically, functional status, evaluation of daily activities of living, and lab work including biomarkers like B-type natriuretic Peptide (BNP) or NT-proBNP are used to predict the likelihood of MINS [16]. Further cardiac tests such as electrocardiogram, echocardiography, and stress tests may be conducted in patients with identified cardiovascular risk factors before surgery. As discussed previously, the assessment of cardiac risk indices is imperative to estimate perioperative and postoperative cardiac risk and the necessity of a cardiology consult before surgery.

Additionally, risk factor modification is crucial to improve patient outcomes preoperatively as well as during the postoperative period. Implementing interventions to manage modifiable risk factors in noncardiac surgical patients includes lifestyle modifications such as smoking cessation, a healthy diet, and regular physical activity [16]. Medication management is also critical in preventing MINS. The use of an antihypertensive regimen is useful in reducing the risk of perioperative cardiovascular events, as well as lipid-lowering medications, antiplatelet therapy, and glycemic control in surgical patients [16]. For patients with hypertension, monitoring blood pressure regularly as well as maintaining a healthy weight, reducing sodium intake, and limiting alcohol consumption are all modifiable factors that can reduce cardiovascular complications. Temporary adjustments or discontinuation of medications that involve bleeding risks is another factor to consider before patients undergo surgical procedures [16].

Throughout the preoperative assessment and risk factor identification process, patients and caregivers should be given education regarding the signs and symptoms of cardiovascular complications like MINS and taught the importance of seeking medical care immediately if these symptoms occur. Ultimately, patient education should also include guidance on lifestyle modifications, the importance of medication adherence, and follow-up appointments with primary caregivers and specialists to further optimize long-term cardiovascular health and reduce the risk of MINS. A comprehensive overview of MINS prevention strategies is provided in Table 1.

**Table 1:** MINS prevention strategies

MINS Prevention Strategies		Description	Level of Evidence
Risk assessment tools	RCRI	Calculates risk based on high-risk noncardiac surgery, history of ischemic heart disease, CHF, TIA/stroke, preoperative treatment with insulin, and preoperative creatinine over 2 mg/dL	● Used to guide postoperative cardiac monitoring, ● Has limited predictive accuracy of cardiac events/death, especially following vascular non-cardiac surgeries
	ACS-NSQIP	● Offers more individualized risk assessment ● based on over 135 variables ● compares performance to national standards	● Reduction of postoperative complications and mortality by 45% and 27%, respectively ● may have limited predictive accuracy and surgical bias^13^
	Gupta Perioperative Risk for MICA	● Similar to RCRI but more accurate at identifying high-risk patients ● Does not overestimate low-risk population ● Uses parameters specific to cardiac injury to determine risk of developing MINS	● Factors in various surgery types, including minimally invasive, and differentiates them in the context of intra-operative and post-operative MI or cardiac arrest^13^ ● Retrospective data likely underestimates amounts of MI reported
	VISION risk calculator	● Uses the same risk factors assessed in RCRI as well as NT-proBNP levels to predict cardiovascular injury within 30 days post-operation	● Provides updated estimates to the RCRI assessment by evaluating perioperative cardiovascular risk up to 30 days after surgery ● Limitation of external validation regarding NT-proBNP thresholds within other patient cohorts^14^
Minimize cardiovascular risk factors	Utilize preoperative cardiac assessment by evaluating functional status, evaluation of daily activities of living, and lab work specifically biomarkers like BNP or NT-proBNP and electrocardiogram, echocardiography, and stress tests if indicated		
Risk factor modification	Lifestyle modifications include smoking cessation, a healthy diet, and regular physical activity Medication management for other comorbid conditions such as hypertension and diabetes mellitus		
Patient and provider education	Healthcare providers can impart guidance on lifestyle modifications, the importance of medication adherence, and follow-up appointments with primary caregivers and specialists		

### Intraoperative and perioperative interventions for mins

During the intraoperative period, the patient care team must monitor for early detection of myocardial injury, evaluate anesthetic considerations, maintain hemodynamic stability, and minimize stress on the cardiovascular system. This can include optimizing fluid balance, ensuring pain control, and using appropriate anesthetic techniques to minimize cardiac stress. To optimize intraoperative and perioperative intervention for MINS during noncardiac surgical procedures, a multidisciplinary approach involving the collaboration between surgeons, anesthesiologists, cardiologists, and internal medicine providers are ideal to ensure proper management and coordination of interventions.

### Monitoring techniques for early detection of myocardial injury

Assessing myocardial injury through the use of intraoperative monitoring techniques is critical for preventing and managing myocardial injury after noncardiac surgery. These techniques include continuous electrocardiogram monitoring, invasive arterial blood pressure monitoring, transesophageal echocardiography, biomarker monitoring, near-infrared spectroscopy, pulse oximetry, and central venous oxygen saturation [17]. Further, intraoperative imaging techniques such as magnetic resonance imaging or computed tomography angiography are critical for assessment of myocardial perfusion or the detection of acute coronary artery occlusions.

Monitoring techniques are important for numerous reasons. Early detection of myocardial injury or ischemia is crucial for prompt intervention and prevention of adverse outcomes such as cardiac arrest [17]. Continuous monitoring also allows for identifying patients at higher risk of developing cardiac complications, which in turn provides closer surveillance in high-risk patients undergoing noncardiac procedures [17]. Information gained from cardiac function monitoring techniques may guide intraoperative management enabling the optimization of hemodynamics, oxygen delivery, and tissue perfusion during surgery [17]. When intraoperative interventions are aimed towards preventing or managing myocardial injury during surgery, monitoring techniques allow clinicians to continuously assess the patient and guide adjustments in care. Overall, monitoring techniques during the perioperative period play a crucial role in the early detection, risk stratification, and management of MINS by providing continuous assessment of cardiac function. This enables healthcare providers to provide timely intervention and optimization of care, ultimately improving outcomes for surgical patients at risk of developing myocardial injury after noncardiac surgery.

### Anesthetic considerations and hemodynamic management strategies

The anesthetic considerations and hemodynamic management during surgery focuses on optimizing perioperative hemodynamics, minimizing myocardial oxygen demand, avoiding hypotension, and optimizing cardiac output to reduce the risk of myocardial injury.

Regarding perioperative hemodynamics, fluid management is based on specific patient characteristics and intraoperative needs. However, the use of balanced crystalloid solutions may help anesthesiologists avoid fluid overload and further exacerbate myocardial injury [18]. Additionally, during surgery, vasoactive agents may need to be utilized in order to maintain an adequate mean arterial and perfusion pressures. Overall, hemodynamic optimization strategies are geared towards parameters such as maintaining stroke volume, and pulse pressure variation which aids in guiding fluid resuscitation and vasopressor use to avoid excessive fluid administration.

When utilizing intraoperative anesthetics, it is imperative to minimize the use of medications that have adverse effects of depressing myocardial function, such as certain volatile anesthetics and opioids [19]. Using regional anesthesia techniques such as epidural or spinal anesthesia in appropriate situations may provide better hemodynamic stability and reduce the need for systemic opioids during surgery; although, this has not been confirmed to prevent MINS [18]. Additionally, choosing anesthetic agents that minimize myocardial depression such as volatile anesthetics with cardioprotective properties like sevoflurane or total intravenous anesthesia techniques with agents like propofol and remifentanil, should be preferred when preventing cardiac complications such as MINS [19]. However, there has not been an identified link between anesthesia agent types and the prevention or development of MINS [18]. In 2014, the Evaluation of Nitrous Oxide in the Gas Mixture for Anesthesia trial (ENIGMA-II) led by Myles and associates analyzed noncardiac surgical patients and the use of nitrous oxide during surgery [20]. The ENIGMA-II trial found that nitrous oxide use during the intraoperative period did not increase the risk of developing MINS [20]. Overall, future studies geared towards anesthetic agents and their association with developing MINS is necessary for preventing this common cardiac complication.

Avoiding excessive vasodilation and hypotension is a necessity during the intraoperative period as these conditions can worsen myocardial injury. According to Salmasi and associates, there is a potential association between intraoperative hypotension and the development of MINS [21]. A study conducted by Maheshwari found that continuous noninvasive blood pressure monitoring during noncardiac surgery halved the amount of hypotensive episodes which may contribute to the development of MINS [22].

For maintaining tissue perfusion and oxygen delivery during surgery, cardiac output must be normalized and maintained. To do so, ventilator optimization, positioning, and temperature management are all key to maintaining cardiac output. Using lung-protective ventilation strategies to minimize barotrauma and other types of ventilator-induced lung injury can help prevent changes to cardiac output [18]. Also, Trendelenburg position or leg elevation can optimize cardiac preload and venous return when needed for specific surgical sites and noncardiac surgeries. Maintaining intraoperative normothermia is also imperative to preventing cardiac function impairment and increasing the risk of adverse cardiovascular events [18]. Lastly, if myocardial dysfunction occurs during noncardiac surgery, inotropic support can improve myocardial contractility and cardiac output but must be used cautiously to avoid dysrhythmic effects [18].

## Long-term management of mins

### Postoperative monitoring and follow-up

Postoperative care aimed at preventing and managing MINS involves a combination of monitoring, medical management, and supportive interventions. For instance, continuous monitoring of vital signs including heart rate, blood pressure, and oxygen saturation can detect signs of cardiac compromise or ischemia in a postoperative setting. ECG monitoring in high-risk patients with preexisting cardiac conditions can also promptly assess for rhythm disturbances or provide evidence for myocardial ischemia [22]. Adequate pain control using multimodal analgesia techniques to minimize sympathetic activation may prove useful in reducing the risk of perioperative myocardial ischemia that leads to MINS. It is also important to avoid opioid overuse, as this can lead to respiratory depression and hemodynamic instability. Postoperative fluid management may help the patient maintain euvolemia and prevent fluid overload or dehydration, which can exacerbate cardiac strain and compromise cardiac perfusion [22]. Another priority includes the administration of antihypertensive medications to meet target blood pressure. Strict blood pressure control postoperatively helps to minimize the cardiac workload as well as reduce the risk of perioperative myocardial ischemia [22]. Lastly, early mobilization and thromboprophylaxis are key components of preventing cardiovascular complications in the postoperative setting. Early ambulation helps to prevent venous stasis, thromboembolic events, and deconditioning which all contribute to cardiovascular complications. Preventing deep vein thrombosis and pulmonary embolism postoperatively is of utmost importance as these pathologies can pose significant cardiovascular threats after surgical intervention.

### Pharmacological management for secondary prevention

Post-operative management includes close monitoring for signs of myocardial injury, with a focus on early detection and treatment of complications. This may involve the use of medications to manage symptoms and prevent further cardiac damage, such as aspirin, statins, anticoagulants, angiotensin-converting enzyme (ACE) inhibitors, beta-blockers, and DAPT depending on the patient’s specific risk factors and clinical presentation.

The role of aspirin in the prevention and treatment of MINS remains controversial. While aspirin can be given to prevent postoperative thrombosis formation, results from the POISE-2 trial did not support the conclusion that aspirin improves mortality rates associated with MINS within 30 days [19]. However, postoperative aspirin has been found to decrease the risk of major cardiac events and is still regularly given by providers as prophylactic anticoagulation after major surgeries [23]. Overall, aspirin therapy should be carefully considered by physicians and be based on individual patient’s risk factors and bleeding risk.

New research has surmised that statins may play a critical role in the management of MINS by reducing the likelihood of cardiovascular events. Since a major risk factor for developing MINS is atherosclerotic disease, the ability of statins to lower cholesterol and stabilize arterial plaques may be beneficial for reducing mortality rates. According to Park, patients diagnosed with MINS who were treated with statins 1-year postoperatively had significantly lower mortality rates than patients in the no-statin group [23]. While more research is needed to fully explore the usability of statins for MINS patients, statins continue to play a crucial role in reducing cardiovascular events in postsurgical patients by promoting vascular health.

ACE inhibitors play an important role in the management of MINS by controlling blood pressure, improving endothelial function, and providing cardioprotective and anti-inflammatory benefits [23]. However, withholding ACE inhibitors 24 h before major noncardiac surgery was associated with a lower risk of death and other postoperative complications [24]. While ACE inhibitors may improve circulation and reduce the risk of complications in MINS, there is no concrete data available regarding its benefit in treating MINS postoperatively.

The POISE Trial emphasized that beta blockers, namely metoprolol, prevented perioperative ischemic events but increased the overall mortality of patients undergoing noncardiac surgeries [25]. It is hypothesized that beta-blockers may increase mortality rates due to increasing the risk of hypotension, causing ischemic changes leading to MINS. The MANAGE trial found that dabigatran significantly lowered the risk of major vascular complications in patients with MINS, suggesting its potential benefit for reducing cardiovascular events [26]. Specifically, dabigatran 110 mg twice daily was theorized to aid MINS patients by reducing their mortality rates and risks of developing major cardiovascular events [26]. The role of anticoagulation therapy in MINS remains an ongoing debate, as some studies have argued that there is no significant benefit of utilizing anticoagulation therapy in patients with MINS. The management of DAPT in the context of non-cardiac surgery is complex, with guidelines recommending elective non-cardiac surgery to be performed 6 months after DES placement, and in urgent surgeries, continuing DAPT with minimal interruption of aspirin. Further, the use of aspirin and a purinergic receptor type y, subtype 12 inhibitor has not been routinely recommended for the management of MINS. While DAPT therapy is the standard treatment to prevent recurrent ischemic events in patients with acute coronary syndrome, the benefit of DAPT in MINS is unclear [26]. MINS is characterized by a myocardial injury that occurs in the absence of acute coronary syndrome, however, both may be treated with antiplatelet agents, anticoagulants, and statins to prevent recurrent ischemic events.

### Lifestyle modifications and cardiac rehabilitation programs

Patients with MINS may require long-term management of cardiovascular risk factors, including lifestyle modifications and adherence to a medication regimen to prevent future cardiac events. Certain conservative management techniques can be recommended by healthcare providers such as smoking cessation, a balanced diet, and moderate physical activity. It is also imperative to optimize the management of other comorbidities such as hypertension and diabetes in patients who are at risk for developing MINS.

Cardiac Rehabilitation (CR) has been shown to reduce cardiac-related mortality and hospitalizations [27]. Used primarily as a form of tertiary prevention, CR encompasses both a tailored, physician-supervised exercise program and counseling on lifestyle modification in patients with coronary artery disease, heart failure, and valvular disease. Therefore, this method suggests a potentially beneficial role in patients with MINS. However, this method could create more opportunities for necessary investigation as limited data on the benefits of cardiac rehabilitation in patients with MINS currently exists [27].

## Challenges and future directions

### Limitations of current prevention and management strategies for mins

While there has been significant research on the topic of MINS in more recent years, several limitations continue to persist. For instance, the lack of routine cardiac biomarker monitoring in noncardiac surgery patients makes it difficult for clinicians to promptly diagnose and greatly delays management. In addition, the specific prevention and management protocol is in contention within the research community, as more research is needed to identify the most effective strategies in treating this condition. Another limitation includes the heterogeneous patient population which encompasses a spectrum of health risks, comorbidities, and perioperative management strategies which makes it challenging to diagnose and treat MINS. Lastly, the optimal timing for initiating pharmacological therapies is not well-defined. Determining the timing and duration of pharmacological interventions requires further studies and analyses.

### Emerging technologies and interventions for improving outcomes

Several emerging technologies show promise for improving outcomes in MINS. Firstly, high-sensitivity troponin assays allow for earlier and more sensitive detection of myocardial injury [9]. Noncardiac surgical patients do not routinely require cardiac biomarker assays, so utilizing these assays may enable prompt intervention for MINS patients as well as provide care providers with more sound risk stratification. Further, biomarkers related to inflammation and oxidative stress are being investigated as prevention and intervention techniques for treating those with MINS [9]. Lastly, enhanced recovery after surgery protocols that focus on reducing surgical stress and optimizing perioperative care may help reduce the incidence and severity of MINS by decreasing oxidative insult during surgery [28].

Many common diseases demonstrate a genetic susceptibility. As genetic testing becomes more widely available, we may see that it can significantly contribute to identifying at-risk patients. One recent retrospective study by Douville and associates has already proposed to consider the revision of some of the MINS risk assessment tools to include a genetic variable [29]. In this study, investigators found that incorporating a polygenic risk score incrementally improved the ability to predict MINS. However, it should be noted that this study had limited prospective identification of at-risk patients and could not determine if this identification would prompt clinically relevant management changes [29].

## Conclusion

### Summary of key findings

The management of MINS requires a multifaceted approach that includes early detection, risk stratification, preventive measures, and both acute and long-term management strategies. An interdisciplinary team is crucial in providing comprehensive care to improve outcomes for patients experiencing myocardial injury after noncardiac surgery. Despite the use of aspirin, statins, ACE inhibitors, and DAPT, mortality and MACE rates remain high, further highlighting the need for ongoing research and tailored management protocols.

### Implications for clinical practice

MINS research has significant implications for clinical practice including the importance of early detection, routine monitoring, the importance of utilizing a multidisciplinary team, and long-term follow-up and rehabilitation. Firstly, studies have emphasized the importance of early detection and risk stratification of MINS using high-sensitivity cardiac troponin assays and other biomarkers [9]. In addition to this, implementing routine monitoring of cardiac biomarkers can enable prompt intervention for high-risk patients. Research has also identified modifiable risk factors as well as specific perioperative management strategies that can reduce the incidence and mortality risk associated with MINS [16]. The data associated with MINS continuously emphasizes the importance of multidisciplinary collaboration between surgeons, anesthesiologists, cardiologists, and primary care providers as coordinated care is essential for optimizing patient outcomes and preventing cardiac complications. Additionally, long-term follow-up and cardiovascular rehabilitation programs are imperative for optimizing cardiovascular health. Further, structured rehabilitation programs can assist in improving outcomes and patient quality of life. While there is no denying that MINS has become a popular topic of research within the medical community since it was first described in 2014 and with inclusion in the well-known VISION study, one must consider the proposal to expand diagnostic criteria that could aid in earlier detection of otherwise undiagnosed MINS cases and continue to explore better preventative and interventional measures. With much of the data still in its infancy, more investigation is justified considering this relatively common phenomenon that continues to impact healthcare worldwide.

## Data Availability

No datasets were generated or analysed during the current study.

## Abbreviations

MINS: Myocardial Injury after Noncardiac Surgery
DAPT: Dual Antiplatelet Therapy
MACE: Major Adverse Cardiac Event
BNP: B-Type Natriuretic Peptide
Nt-ProBNP: N-terminal Pro-B-type Natriuretic Peptide
GDF-15: Growth Determining Factor-15
VISION: Vascular Events in Noncardiac Surgery Cohort Evaluation
RCRI: Revised Cardiac Risk Index
ACS NSQIP: American College of Surgeons National Surgical Quality Improvement Program
MICA: Gupta Perioperative Risk for Myocardial Infarction or Cardiac Arrest
ECG: Electrocardiogram
ENIGMA-II: Evaluation of Nitrous Oxide in the Gas Mixture for Anesthesia trial
POISE: Perioperative Ischemic Evaluation trial
ACE inhibitors: Angiotensin-converting enzyme inhibitors
MANAGE trial: Dabigatran in patients with myocardial injury after non-cardiac surgery
CR: Cardiac rehabilitation
